# Serum amyloid A is elevated in the serum of lung cancer patients with poor prognosis

**DOI:** 10.1038/sj.bjc.6605700

**Published:** 2010-05-25

**Authors:** W C S Cho, T T Yip, W W Cheng, J S K Au

**Affiliations:** 1 Department of Clinical Oncology, Queen Elizabeth Hospital, 30 Gascoigne Road, Kowloon, Hong Kong; 2 Ciphergen Biosystems Inc, Fremont, CA, USA

**Keywords:** lung cancer, proteinchip, proteomics, SELDI-TOF-MS, serum amyloid A, serum biomarker

## Abstract

**Background:**

Lung cancer is known as the top cancer killer in most developed countries. However, there is currently no promising diagnostic or prognostic biomarker for lung cancer. This study aims to discover non-invasive differential markers in the serum of lung cancer patients, to determine the protein identity of the candidate biomarker(s), and to investigate any clinical implication of the biomarker(s) concerned.

**Methods:**

Blood specimens were collected from 154 pre-operative patients with lung cancer and 35 healthy blood donors with no evidence of lung cancer. Fractionated serum samples were processed by surface-enhanced laser desorption/ionisation time-of-flight mass spectrometry (MS). Candidate biomarker was identified using sodium dodecyl sulphate polyacrylamide gel electrophoresis and tryptic digestion followed by tandem MS fragmentation analysis, which was subsequently validated with immunoassay.

**Results:**

A differential protein with *m/z* 11.6 kDa was detected and identified as an isoform of human serum amyloid A (SAA). It was significantly increased by 1822% in lung cancer patients when compared with the healthy controls, which gave an area under the receiver operator characteristic curve of 0.88. In addition, the protein was also significantly elevated by 77% in lung cancer patients with survival <5 years when compared with patients with survival ⩾5 years.

**Conclusion:**

There are several functions of the SAA protein, described in the context of inflammation, that are compatible with the mechanism of tumour invasion and metastasis. Our study not only detected increased SAA level in the serum of lung cancer patients but also identified that elevated SAA level may be a non-invasive biomarker useful for the prediction of lung cancer prognosis.

Lung cancer is known as the top cancer killer in most developed countries. Despite marked progress in the treatment, most patients still die of the disease within several months to a few years. The majority of lung cancer is diagnosed at a relatively late stage, and recurrence is common even after optimal therapy. However, there is currently no promising diagnostic or prognostic biomarker for this lethal disease (Cho, 2007a, 2010a). Proteins are the workhorse molecules of life. As proteome changes in response to cancer, it is of great interest to medical researchers. There is hope that oncoproteomics research may help to develop new approaches for early cancer diagnosis, to determine the best therapies for individual patients with specific types of cancer, and to predict whether cancer will recur after treatment (Cho and Cheng, 2007; Cho, 2007b; Cohen *et al*, 2008; Gámez-Pozo *et al*, 2009; Han *et al*, 2009). In recent years, proteomics has been propelled by advances in mass spectrometry (MS) that high-throughput protein profiling enables large-scale screening of proteins within a small sample volume (Cho, 2009). This study aims to use protein profiling for the identification of serum biomarkers that can distinguish patients with lung cancer from healthy controls, as well as differentiate lung cancer patients between poor prognosis and good prognosis, which may potentially provide non-invasive biomarkers for the detection of lung cancer and the prediction of survival in lung cancer patients.

## Materials and methods

### Serum samples

Human sera were obtained through hospital-approved protocols at the Queen Elizabeth Hospital in Hong Kong from each non-fasting individual with provision of informed consent indicating voluntary participation. Specimens were collected from pre-operative patients diagnosed with lung cancer (*n*=154) and blood donors with no evidence of lung cancer (*n*=35). Information was collected from each patient pertaining to age and gender. Healthy controls were selected from an archive of blood samples on the basis of matching for age, sex, minimum previous handling, and time period of collection similar to the lung cancer group. The average age±standard error of the mean (s.e.m.) for the lung cancer patients was 65.5±0.9 years. Among these patients, 83.8% were male (*n*=129) and 16.2% were female (*n*=25). Regarding the stages of the disease, 55% (*n*=85) of the patients were diagnosed with early-stage lung cancer and 45% (*n*=69) were diagnosed with advanced-stage lung cancer. Within the cancer samples, 34.4% were squamous-cell carcinoma samples (*n*=53), 32.5% adenocarcinoma (*n*=50), 8.4% other non-small-cell lung cancer (*n*=13), and 24.7% small-cell lung cancer (*n*=38). A volume of 5 ml of blood sample from each participant was allowed to clot for 30 min to 1 h at room temperature and was centrifuged at 1500 **g** for 10 min. All sera were aliquoted and frozen at −70°C until thawed specifically for protein profiling.

### Sample fractionation

To increase the detection of a larger number of peaks, as well as to alleviate signal suppression effects on low-abundant proteins from high-abundant proteins such as albumin, serum samples were fractionated into six fractions containing proteins separated roughly on the basis of their isoelectric points, as previously described (Cho *et al*, 2007). Briefly, 20 *μ*l of each serum sample was diluted during fractionation in 30 *μ*l of 50 mM Tris-HCl buffer (pH 9) containing 9 M urea and 2% 3-((3-cholamidopropyl)dimethylammonio)-1-propanesulfonate (CHAPS). A further dilution was made in 50 mM Tris-HCl buffer (pH 9) containing 1 M urea and 0.22% CHAPS. Serum samples were loaded onto each well of a 96-well filter plate pre-filled with a Q Hyper DF anionic exchange sorbent (Ciphergen Biosystems, Fremont, CA, USA) and eluted in a stepwise pH gradient using a Biomek 2000 liquid-handling robot (Beckman Coulter, Fullerton, CA, USA), as described by the manufacturer. The fractions contained the flow-through proteins eluted with pH 7, 5, 4, and 3, and an organic buffer. Each of the six fractions was collected twice and the two collections were pooled. This helped to ensure the pH changes appropriately and also gave greater reproducibility in the fractionation, as well as better partitioning of proteins into their respective fractions.

### Protein profiling

Fractioned serum samples were processed for surface-enhanced laser desorption/ionisation time-of-flight (SELDI-TOF) MS (Ciphergen Biosystems) analysis using the eight-spot weak cation exchange (WCX2) ProteinChip (Ciphergen Biosystems) as previously described (Au *et al*, 2008). Each sample was assayed in duplicate, having duplicate samples randomly placed on different proteinchips with the aid of a 96-well bioprocessor. A pooled quality control sample prepared in the same manner was applied to duplicate spots on each proteinchip used in the experiment as a reproducibility control. To equilibrate the proteinchips, 150 *μ*l of binding buffer (100 mM sodium acetate at pH 4) was added into each well. The bioprocessor was sealed and incubated with the samples at room temperature for 5 min, with vigorous agitation on a MicroMix 5 shaker (Diagnostics Products Corporation, NJ, USA). The buffer was removed after vortexing. To bind samples onto the proteinchips, 90 *μ*l of binding buffer (100 mM sodium acetate at pH 4) and 10 *μ*l of fractioned sample was added into each well and vortexed for 30 min at room temperature. A further 150 *μ*l of binding buffer (100 mM sodium acetate at pH 4) was added into each well and vortexed for 5 min at room temperature. The excess sera mixtures were discarded after vortexing. The proteinchips were washed twice with de-ionised water and then removed from the bioprocessor. A saturated solution of 1 *μ*l sinapinic acid (Ciphergen Biosystems) in 50% (v/v) acetonitrile and 0.5% (v/v) trifluoroacetic acid was applied twice to each spot and air dried for 10 min between the applications. The proteinchips were air-dried and analysed using a proteinchip reader PBS-IIc (Ciphergen Biosystems) with acquisition up to 200 kDa. Spectra were collected by the accumulation of 232 shots at laser intensity 180 in a positive mode. The protein masses were calibrated externally using the all-in-one peptide standard (Ciphergen Biosystems).

### Protein identification

The differential protein of interest between the cancer sera and healthy controls was identified with sodium dodecyl sulphate polyacrylamide gel electrophoresis (SDS-PAGE) and tryptic digestion followed by tandem MS fragmentation analysis. Proteins fractionated at organic solvent eluant from the anion exchange filters were characterised by SDS-PAGE. The protein band containing the desired biomarker was cut from the gel and subjected to overnight tryptic digestion. Protein identification was confirmed by tandem MS fragmentation analysis of the peptide generated from the tryptic digest. Tandem MS fragmentation of the peptide generated a set of tandem MS ion fingerprint data, which was exported for database matching and subsequent identification using the Mascot program (Matrix Science Inc, Boston, MA, USA).

#### Quantitative analysis of serum samples for serum amyloid A

The concentration of serum amyloid A (SAA) was determined in both the lung cancer sera (*n*=38) and the healthy control sera (*n*=34) using a commercially available immunoassay kit (BioSource, Brussels, Belgium), and the analysis was performed according to the manufacturer's instructions.

### Statistical analysis

The CiphergenExpress software version 3.0.6 (Ciphergen Biosystems) was used to compare serum protein profiles to discover peaks that were differentially expressed between two comparable groups. To visualise the protein peaks that differed in the majority of samples in one group compared with the majority of samples in the other, principal components analysis (PCA) was performed to reduce the dimensionality of the data set and to identify new meaningful underlying variables with visualisation by three-dimensional PCA display. Hierarchical clustering was also conducted and visualised by heat map, a graphical representation of the entire array in grid form with columns representing samples and rows showing the mass-to-charge ratio (*m/z*) of protein peaks. For univariate analysis, the average normalised intensity of each peak was calculated for the comparable groups. The difference in group means was reported as the percentage of change, that is, the percentage of (average intensity of one group − average intensity of the compared group)/average intensity of the compared group. The comparison of *P*-values was analysed by the non-parametric Mann–Whitney *U*-test; a peak was deemed to show a statistically significant difference in group means if its *P*-value was <0.05. In addition, the receiver operator characteristic analysis of each protein peak was performed for the comparable groups, with the area under the curve value calculated for each peak.

## Results

### Serum protein profiling in lung cancer patients

A total of 245 peaks were detected with *m/z* ranging from 1 to 197 kDa. Of these, 53 peaks were generated from fraction 1 (pH 9 and flow through), 43 peaks were generated from fraction 2 (pH 7), 28 peaks were generated from fraction 3 (pH 5), 38 peaks were generated from fraction 4 (pH 4), 39 peaks were generated from fraction 5 (pH 3), and 44 peaks were generated from fraction 6 (organic eluant).

### Detection of lung cancer

**Figure 1:**
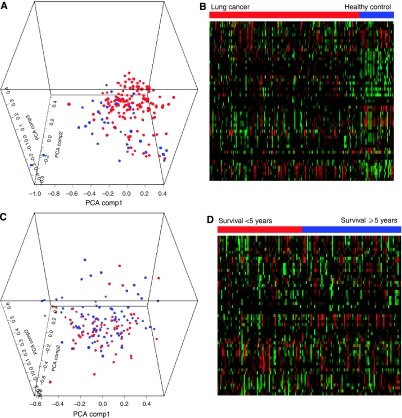
Multivariate analysis: three-dimensional principal components analysis (PCA) and hierarchical clustering analysis (red, upregulated; green, downregulated). (**A**) PCA for healthy control (*n*=35) *vs* lung cancer (*n*=154). (**B**) Hierarchical clustering analysis for healthy control (*n*=35) *vs* lung cancer (*n*=154). (**C**) PCA for patients with survival ⩾5 years (*n*=83) *vs* patients with survival <5 years (*n*=71). (**D**) Hierarchical clustering analysis for patients with survival ⩾5 years (*n*=83) *vs* patients with survival <5 years (*n*=71).

Comparison of the protein profiles using three-dimensional PCA (Figure 1A) and hierarchical clustering analysis (Figure 1B) showed that the serum profiles of patients with lung cancer (*n*=154) could be separated from the healthy control group (*n*=35). There were 140 peaks with significant discriminatory value at distinguishing the lung cancer sera from the healthy control sera. The 10 most discriminating peaks were shown in Table 1A.

### Predicting survival

To discriminate the good prognosis group (survival ⩾5 years; *n*=83) from the poor prognosis group (survival <5 years; *n*=71), the three-dimensional PCA (Figure 1C) and hierarchical clustering analysis (Figure 1D) were performed. There were 24 peaks with significant discriminatory value at distinguishing the good prognosis group from the poor prognosis group. The 10 most discriminating peaks were shown in Table 1B.

### SAA was identified as differential protein

**Figure 2:**
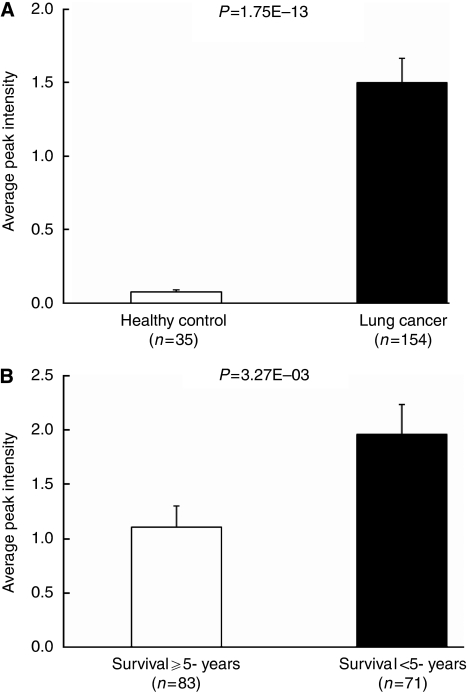
Univariate analysis. (**A**) Bar chart of potential diagnostic biomarkers at *m/z* 11.6 kDa for healthy control (*n*=35) *vs* lung cancer (*n*=154). (**B**) Bar chart of potential prognostic biomarkers at *m/z* 11.6 kDa for patients with survival ⩾5 years (*n*=83) *vs* patients with survival <5 years (*n*=71).

The overexpression of a protein peak at 11.6 kDa was identified as being significantly differentially expressed in both the diagnosis and prognosis analysis. Two bar charts showed the average normalised intensity of this peak's overexpression in the lung cancer sera (*n*=154) when compared with the healthy control sera (*n*=35) (Figure 2A), and the overexpression in the poor prognosis sera (*n*=71) when compared with the good prognosis sera (*n*=83) (Figure 2B). The average normalised intensity value was 19.2-fold higher (*P*=1.75E−13) in the lung cancer sera compared with that in the healthy control sera, and the average expression of the sera from the poor prognosis group was 1.8-fold higher (*P*=3.27E−03) than the sera from the good prognosis group. Using SDS-PAGE and tryptic digestion followed by tandem MS fragmentation analysis, the differential protein at 11.6 kDa was identified as an isoform of human SAA.

### SAA validation by immunoassay

**Figure 3:**
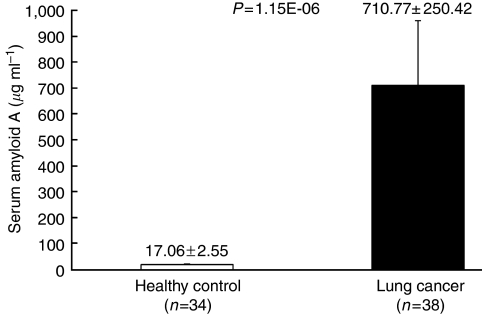
Quantitative analysis of serum samples for serum amyloid A. Bar chart of validation by immunoassay on serum amyloid A levels for healthy control (*n*=34) *vs* lung cancer (*n*=38).

To validate the peak intensity on the SELDI-TOF-MS protein profile with absolute concentrations determined by routine method, the SAA concentration was measured by an immunoassay kit in samples of both the lung cancer sera (*n*=38) and the healthy control sera (*n*=34). The average SAA level was 41.7-fold higher (*P*=1.15E−06) in the lung cancer sera compared with that in the healthy control sera (Figure 3).

## Discusssion

In the omics era, the development of high-throughput technologies that permit the solution of deciphering cancer from higher dimensionality provides a knowledge base that changes the face of cancer understanding and therapeutics (Cho, 2010b). Serum is one of the most easily procured patient specimens and it is perceived to contain many of the molecules that might indicate systemic function. Thus, serum is the sample source that is most often profiled in the hopes of identifying sets of biomarkers for clinical use (Gilbert *et al*, 2004).

In this study, proteomic analyses of serum samples from patients with lung cancer and controls have identified SAA as an elevated biomarker in lung cancer (as shown by SELDI analysis and confirmed by immunoassay). It is known that SAA is secreted during the acute phase of inflammation; the conservation of this protein throughout invertebrates and vertebrates suggests that SAA has an essential role in all animals including humans (Manley *et al*, 2006). There are several functions of SAA protein, described in the context of inflammation, that are compatible with the mechanism of tumour invasion and metastasis. These properties place SAA as an extracellular matrix-associated adhesion protein with a potential role in tumour pathogenesis. The SAA mRNA expression in epithelial cells was gradually increased as they progressed through different stages of dysplasia to overt carcinoma (Gutfeld *et al*, 2006). Accumulating evidence has suggested that SAA might be used to detect a pattern of physiological events that reflect the growth of malignancy and host response. Elevated SAA may be a primary product of tumour lesions, but can also be the product of hepatocytes. Further investigation to determine whether cancer tissue-derived cytokines stimulate SAA synthesis in liver or epithelial cells will be interesting (Malle *et al*, 2009).

Overexpression of SAA has been reported in nasopharyngeal, renal, gastric, hepatocellular, melanoma, breast, and endometrial cancers (Cho *et al*, 2004; Tolson *et al*, 2004; Chan *et al*, 2007; He *et al*, 2008; Findeisen *et al*, 2009; Pierce *et al*, 2009; Ramankulov *et al*, 2008; Cocco *et al*, 2009, 2010; Sasazuki *et al*, 2010; Vermaat *et al*, 2010). Our study not only detected increased SAA level in the serum of lung cancer patients but also identified that elevated SAA level may be a non-invasive biomarker, useful for the prediction of lung cancer prognosis. As far as we know, the association of SAA with the survival of lung cancer patients has never been reported. Moreover, a number of SELDI peaks were found to be significantly differentiated between the serum of cancer patients and controls, as well as between the poor prognosis patients and good prognosis patients. Further identification of these peaks is awaiting.

Recently, the US Food and Drug Administration has approved an ovarian cancer triage test called OVA1, containing CA125 and four biomarkers (*β*_2_-microglobulin, transferrin, apolipoprotein A1, and transthyretin) identified by SELDI-TOF-MS (Fung, 2010). This marks an encouraging step of translating biomarker discovery from laboratory to clinic. It showed that having identified biomarkers by primary screening, a standard assay may then be developed and finally converted into clinically applicable measures. Our results reveal that the level of SAA is highly elevated in lung cancer and in the patients with poor prognosis, thus warranting further studies investigating this candidate biomarker as part of a multimarker test for the diagnosis and prognosis of lung cancer.

## Figures and Tables

**Table 1:** The top 10 peaks best associated with diagnosis and prognosis ranked by *P*-value

**Sample group**	**Peak (*m/z*)**	**Fraction**	**Average peak intensity±s.e.m.**			**Change**		*P*-value*	**AUC**
(A)									
Diagnostic biomarker: healthy control	11 736	Organic	0.109±0.033	*vs*	1.872±0.212	1617%	Up	7.02E−15	0.89
(*n*=35) *vs* lung cancer (*n*=154)	11 563	Organic	0.078±0.012	*vs*	1.499±0.167	1822%	Up	1.75E−13	0.88
	51 215	pH 4	0.327±0.010	*vs*	0.451±0.008	38%	Up	3.12E−11	0.84
	2789	pH 9	1.942±0.331	*vs*	5.418±0.318	179%	Up	2.99E−09	0.80
	7774	Organic	1.247±0.094	*vs*	2.115±0.069	70%	Up	5.79E−09	0.79
	5346	pH 7	1.251±0.182	*vs*	2.896±0.152	131%	Up	1.09E−08	0.79
	66 646	pH 4	13.623±0.217	*vs*	15.204±0.122	12%	Up	3.85E−08	0.77
	17 402	Organic	2.567±0.131	*vs*	1.664±0.056	35%	Down	5.12E−09	0.82
	133 208	Organic	0.302±0.019	*vs*	0.175±0.007	42%	Down	1.66E−09	0.82
	13 918	Organic	1.133±0.068	*vs*	0.722±0.026	36%	Down	2.66E−08	0.80
									
(B)									
Prognostic biomarker: patients with	11 714	pH 7	1.489±0.206	*vs*	2.766±0.416	86%	Up	2.87E−03	0.63
survival ⩾5 years (*n*=83) *vs* patients	11 563	Organic	1.105±0.193	*vs*	1.959±0.275	77%	Up	3.27E−03	0.63
with survival <5 years (*n*=71)	11 695	pH 4	0.753±0.124	*vs*	1.418±0.236	88%	Up	9.01E−03	0.62
	3892	pH 9	7.200±0.383	*vs*	4.788±0.320	34%	Down	2.07E−05	0.71
	17 319	Organic	1.773±0.074	*vs*	1.411±0.066	20%	Down	1.11E−03	0.65
	3958	pH 9	7.707±0.960	*vs*	4.526±0.719	41%	Down	1.47E−03	0.64
	17 577	pH 4	0.307±0.014	*vs*	0.245±0.015	20%	Down	1.53E−03	0.65
	3160	pH 9	7.795±0.933	*vs*	4.684±0.700	40%	Down	2.18E−03	0.64
	3975	pH 9	7.881±0.937	*vs*	4.243±0.589	46%	Down	6.11E−03	0.62
	4303	pH 9	7.720±0.868	*vs*	5.463±0.855	29%	Down	7.85E−03	0.62

Abbreviations: AUC= area under the curve; *m/z*= mass/charge ratio; s.e.m.= standard error of the mean.

^*^P-value from Mann–Whitney *U*-test between the comparable groups.
